# Entrapment of Pulmonary and Intracardiac Thrombus Through Patent Foramen Ovale: A Case Report

**DOI:** 10.7759/cureus.113300

**Published:** 2026-07-24

**Authors:** Faisal M Siddiqui, Haley Patterson, David Baker, Todd Chapman

**Affiliations:** 1 Intensive Care Unit, Carson Tahoe Regional Medical Center, Carson City, USA; 2 Critical Care Medicine, University of Nevada, Reno, USA; 3 Cardiology, Carson Tahoe Regional Medical Center, Carson City, USA; 4 Cardiac Surgery, Carson Tahoe Regional Medical Center, Carson City, USA

**Keywords:** intracardiac thrombus, patent foramen ovale (pfo), pulmonary emboli, pulmonary thrombectomy, surgical embolectomy

## Abstract

We report a case of a 29-year-old man with no significant medical history presenting with pulmonary embolism (PE). The pulmonary thrombus was extensive and connected to the intracardiac thrombus, which further extended through the patent foramen ovale (PFO) over into the systemic side. We decided to pursue surgical embolectomy, resulting in an excellent outcome for our patient. Other approaches, such as systemic anticoagulation and catheter-directed interventions, were considered but not adopted given the risk of systemic embolization. A bedside echocardiogram before catheter-directed intervention timely guided us to pursue the surgical approach. In considering and discussing current evidence-based approaches, we share the clinical reasoning and special considerations in our approach and decision for choosing a surgical treatment pathway for the management of this patient.

## Introduction

Pulmonary embolism (PE) with thrombus entrapment in a patent foramen ovale (PFO) is rare, with an incidence rate of 3.8%, but constitutes a medical emergency due to its associated risk for systemic embolization [1]. The mortality rate from this paradoxical embolization is approximately 18%, and roughly two-thirds of these deaths occur within 24 hours. PE can elevate right-sided atrial pressure and can precipitate entrapment by augmenting right-to-left shunting through the pre-existing PFO. Stroke risk certainly is of paramount concern from this paradoxical embolism in this situation, and requires emergent intervention [1,2]. Therefore, clinical management must take into account previous risk factors of cerebrovascular events while simultaneously providing emergent interventions and careful consideration of managing stroke should it occur. Risk assessment must also be considered with anticoagulation, systemic thrombolysis, and catheter-directed intervention, as these can theoretically be associated with fragmentation of the clot, thereby further increasing the risk of pulmonary and systemic embolization [1,3].

Current guidelines support the use of advanced therapies over anticoagulation alone, with evidence of clot-in-transit [4]. However, this management mainly focuses on the travel of the clot on the pulmonary side, and there is no clear guideline on management principles when paradoxical embolization is imminent. With the emergent nature of the presentation of a clot entrapped in a PFO, plans for intervention, including surgical or catheter-directed thrombectomy (CDT) with PFO closure, must be determined. With the rarity of occurrence and, as mentioned, a dearth of research evidence, no guidelines currently exist identifying a superior method, highlighting the importance of interdisciplinary and patient-centered approaches in care management. Overall, emergent surgical intervention has been shown to be safe and effective while allowing for simultaneous PFO closure, thereby reducing the risk of paradoxical embolism [1,2]. CDT, although more popular in the management of massive and submassive PE, has not been studied in the setting of patients with PE and PFO [2].

We present here a case where there is a connected intracardiac, inter-atrial septal clot extending into the pulmonary arteries. While managing this patient, we acknowledged the legitimate risk of breaking off the clot as it straddled across the atria with the use of conventional catheter-directed embolectomy and systemic thrombolysis. In this instance, surgical embolectomy was employed as it was deemed to be the safest option.

## Case presentation

A 29-year-old man presented with retrosternal chest pain for five days with associated fatigue, dyspnea on exertion, left upper extremity numbness, and left calf pain. Initial history and physical exam did not reveal any risk factors for venous thromboembolism. Of note, his mother had an ischemic stroke and was discovered to have a PFO. He was tachypneic and had mild tachycardia. His vital signs showed oxygen saturation of 92% on room air, blood pressure of 129/73 mmHg, heart rate of 98 beats per minute, temperature of 36.6°C, and a respiratory rate of 28 breaths per minute. Laboratory testing on admission is presented in hematology (Table 1) and chemistry (Table 2) panels.

**Table 1:** Hematology panel

Test name	Measured value	Reference range	Units
White blood cell	8.7	4.5-11.0	× 10³/μL
Hemoglobin	16.0	13.5-17.5	g/dL
Hematocrit	46.6	41.0-53.0	%
Platelet	197	146-390	× 10³/μL

**Table 2:** Chemistry panel showing hyperglycemia, abnormal initial serum troponin, and D-Dimer values AST: aspartate aminotransferase; ALT: alanine aminotransferase; eGFR: estimated glomerular filtration rate

Test name	Measured value	Reference range	Units
Sodium	138	136-144	mmol/L
Potassium	4.0	3.6-5.1	mmol/L
Chloride	104	102-110	mmol/L
Carbon dioxide	24	22-32	mmol/L
Blood urea nitrogen	16	8-20	mg/dL
Creatinine	1.32	0.6-1.10	mg/dL
eGFR	64	>60	mL/min/1.73 m^2^
Glucose	145	60-99	mg/dL
Anion gap	10	2-11	mmol/L
Calcium	9.8	8.7-10.3	mg/dL
Total protein	7.3	6.4-8.2	g/dL
Albumin	4.2	3.5-4.8	g/dL
Total bilirubin	0.9	0.4-2.0	mg/dL
Alkaline phosphatase	74	38-126	U/L
AST	17	15-41	U/L
ALT	18	14-54	U/L
B-type natriuretic peptide	80	<100	pg/mL
Troponin	51	<14	ng/L
D-Dimer	>10	<0.50	µg/mL

**Figure 1:**
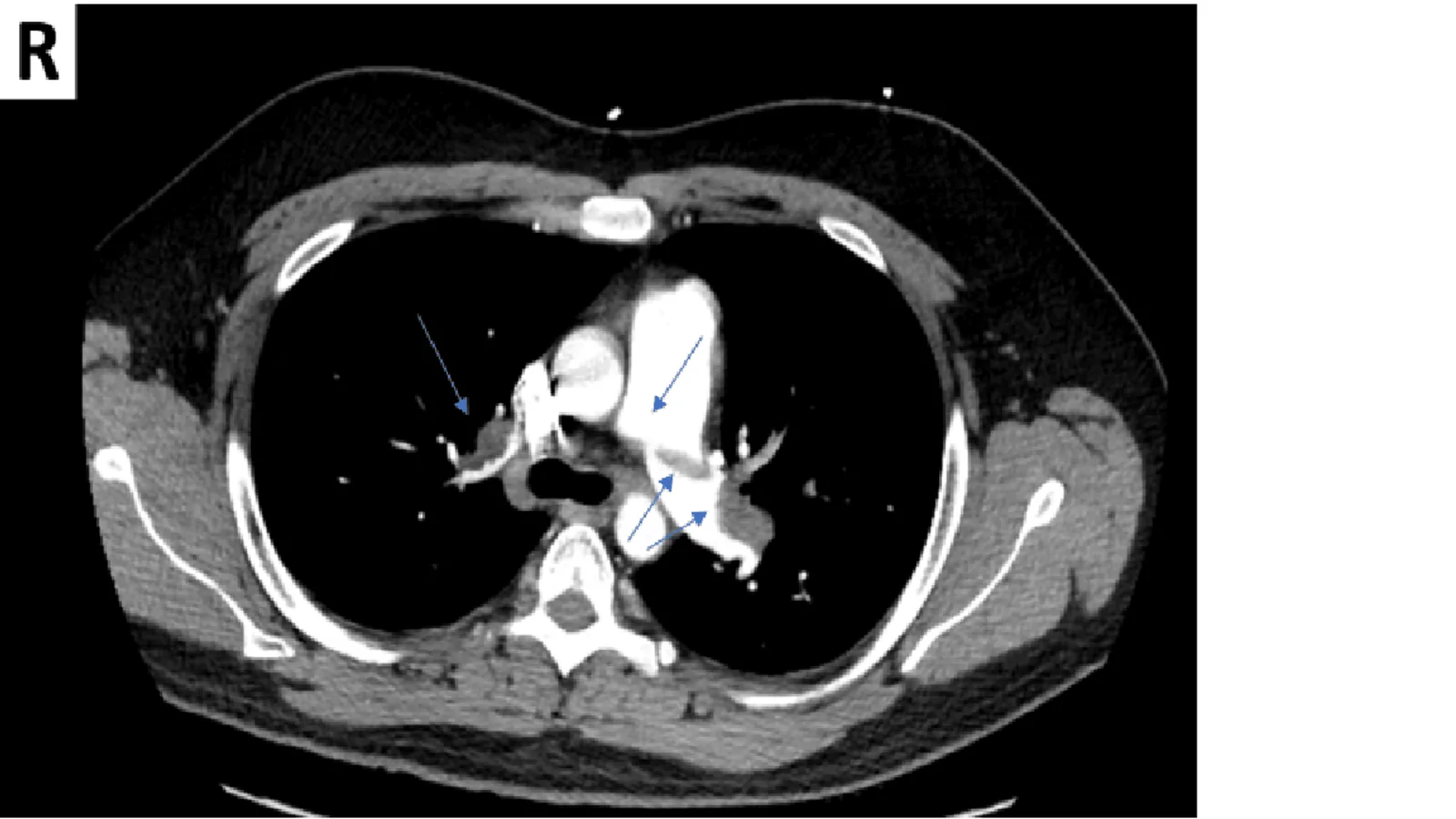
Cross-sectional imaging showing clot burden in the main pulmonary trunk, right and left pulmonary arteries (blue arrows)

**Figure 2:**
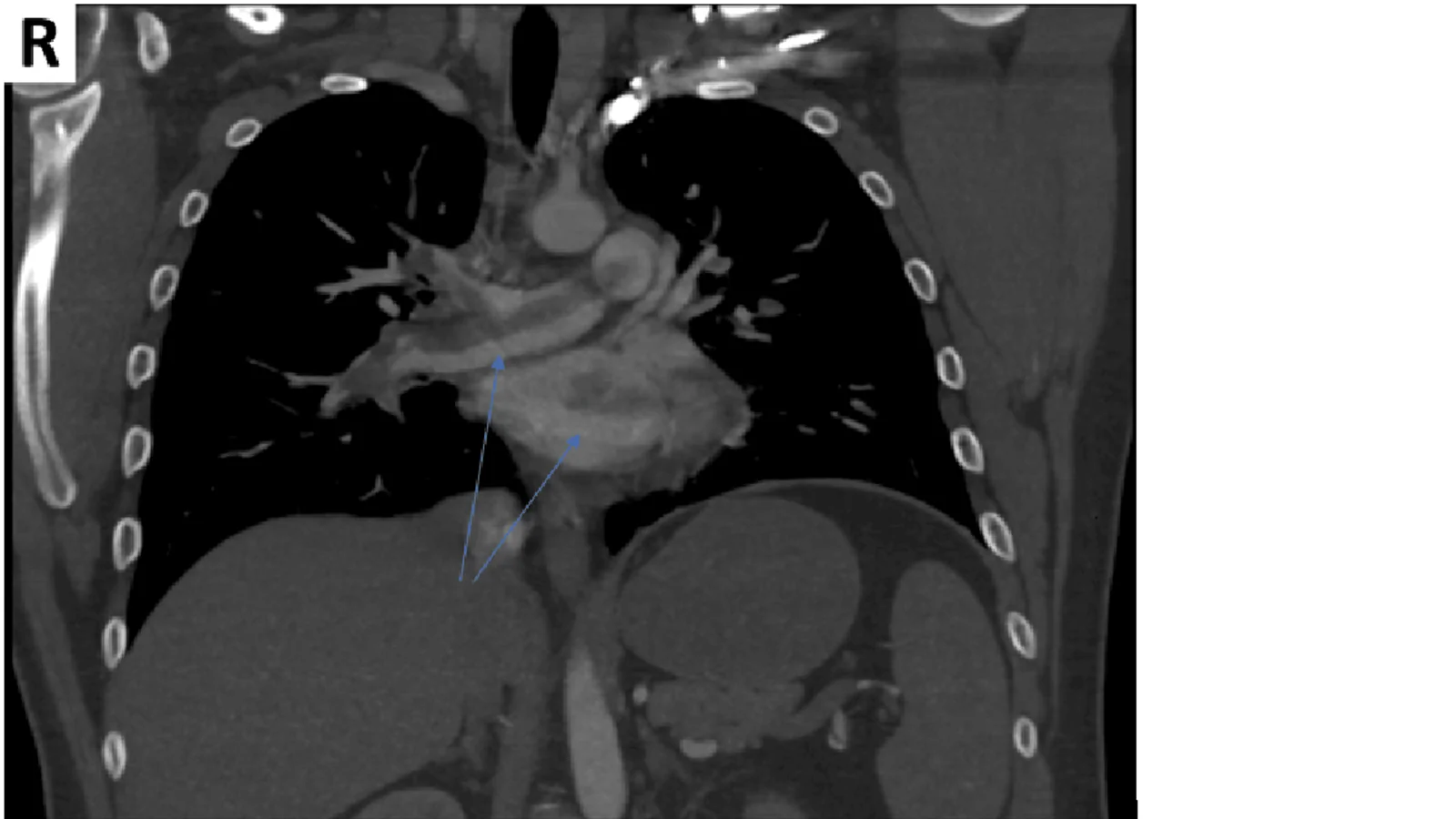
Coronal reconstructed imaging partially showing the extent of the single clot within the pulmonary trunk and the right ventricle (blue arrows)

Pertinent findings were significantly elevated D-dimer at >10 µg/mL (reference range <0.50 µg/mL), increasing serial serum troponin levels (51, 71, 409, 4,627 ng/L; normal<14 ng/L), and normal B-type natriuretic peptide 80 pg/mL (normal<100 pg/mL). Electrocardiography and chest radiography were nonrevealing. Contrast-enhanced computed tomography angiogram of the chest revealed multiple bilateral acute distal pulmonary emboli, and a saddle PE with findings consistent with right heart strain (Figure 1). Given the right ventricular (RV) injury, as reflected by elevated troponin levels and RV dysfunction on cross-sectional imaging, this patient was evidently in the intermediate-high risk category as per the American Heart Association (AHA) classification of PE. Coronal reconstructed images showed the extent of the clot burden in the pulmonary artery and its subsequent branches. A representative image is shown in Figure 2. 

A thrombophilia panel including evaluation for protein C, protein S, factor V Leiden, homocysteine, antithrombin II deficiencies, and anti-cardiolipin/beta-2 glycoprotein antibodies was considered but deferred for a later stage as testing during acute thrombosis and/or while on anticoagulation can distort these results.

**Figure 3:**
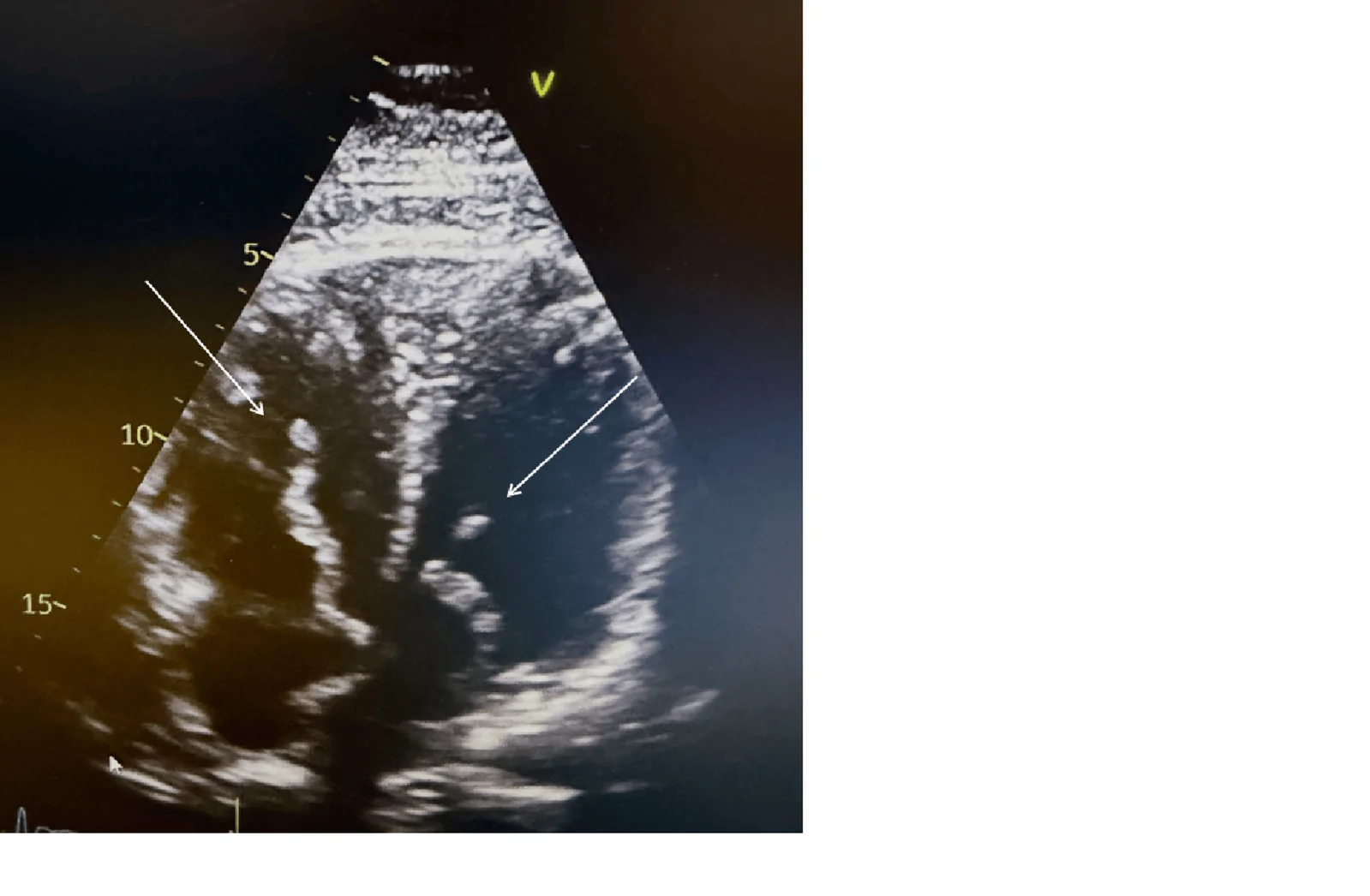
Apical four-chamber echocardiographic image demonstrating the single clot straddling the bilateral atrioventricular (AV) valves. The clot was mobile across both AV valves (arrows pointing at the free ends in each ventricle)

The patient was admitted, and initial plans were made for interventional radiology to proceed with CDT. However, these plans were aborted due to the risk for clot fragmentation, paradoxical embolism, and stroke risk when the primary physician performed a bedside echocardiogram revealing a mobile intracardiac, interatrial clot. This was confirmed with a formal echocardiogram demonstrating a large embolus traversing the PFO, extending from the tricuspid valve through the right atrium across the septal defect through the left atrium and the mitral valve (Figure 3).

**Figure 4:**
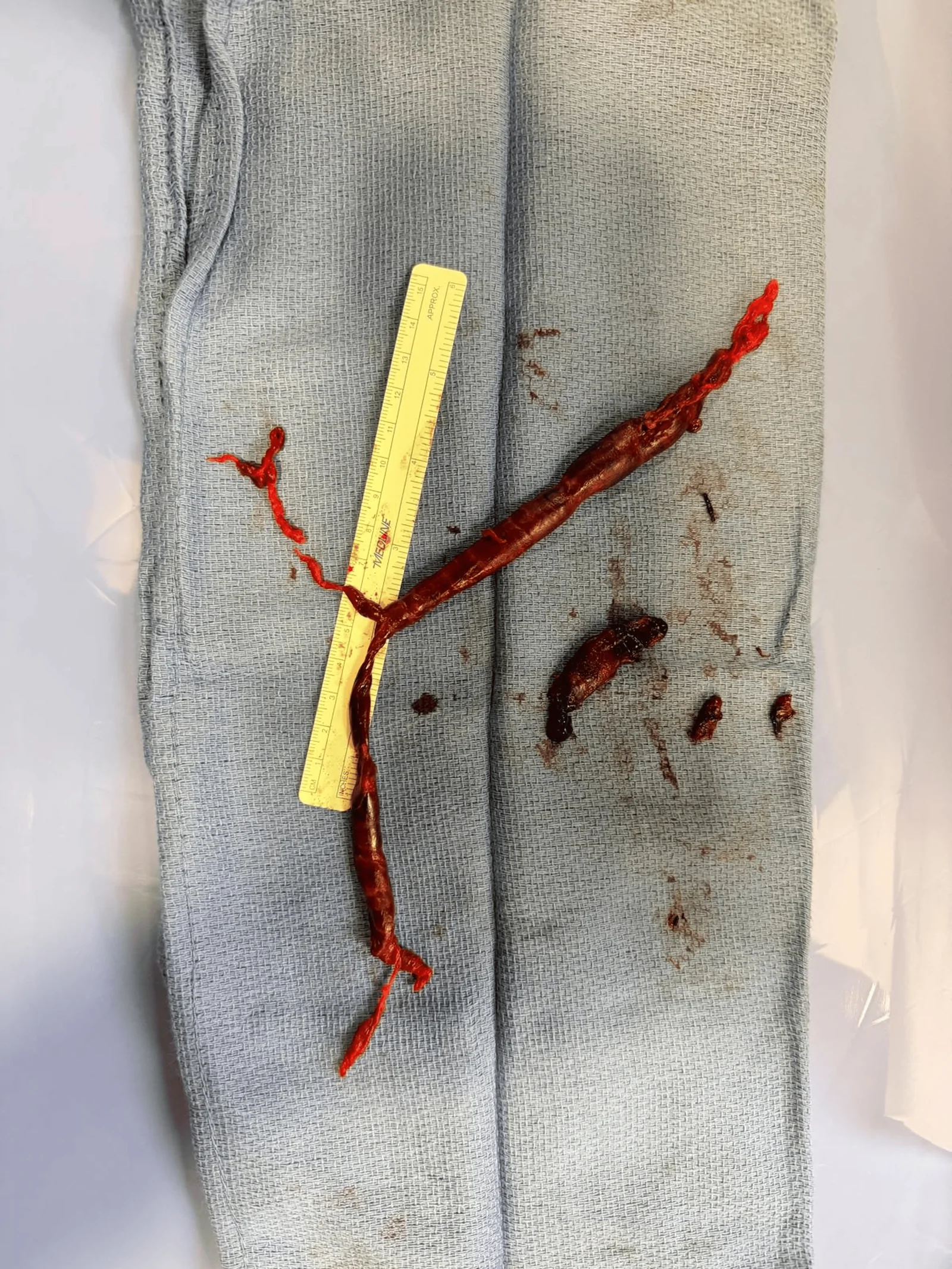
Intraoperative image of the clot that was removed

Systemic anticoagulation was initiated and continued without thrombolysis due to the risk of further clot breakdown and dislodgement. The case was discussed with cardiothoracic surgery as surgical embolectomy was felt to be the only viable option with the least risk of clot fragmentation and stroke risk in a young man. The patient underwent emergent pulmonary embolectomy with simultaneous closure of PFO, intracardiac embolectomy, and pulmonary arteriotomy with thrombectomy, where the clot was removed en bloc with closure of the PFO (Figure 4). A follow-up echocardiogram two days later did not reveal any residual clot; however, it showed persistent RV dysfunction.

Postoperative hypercoagulable workup was completed, including lupus anticoagulant and factor V Leiden mutation analysis, which was unrevealing. The patient was started on Coumadin and was subsequently discharged home.

## Discussion

This case highlights some important points that must be realized at the bedside when managing intermediate-high-risk patients with extensive clot burden in PE. In this patient with PE, interventional radiology was promptly consulted to perform catheter-directed intervention. This approach aligns with the current standard of care, and in a recently published landmark HI-PEITHO trial, it was established that in patients with acute, intermediate-high-risk PE, adding ultrasound-facilitated catheter-directed fibrinolysis improved clinical outcomes [5]. However, the HI-PEITHO trial did not specifically address the issue pertaining to patients with clot-in-transit/PFO. This patient did fit the criteria for inclusion as per the HI-PEITHO trial; however, we argue that if this patient had undergone catheter-directed intervention, there was a potentially devastating risk of breaking off this clot as it straddled across the PFO and bilateral AV valves. The interventional radiologist was unaware of the existence of this PFO, clot size, and extension across the atrial septum into the left atrium and ventricle. Performing a prompt bedside echocardiogram before catheter-directed intervention identified this extensive clot and thereby prompted expedited surgical intervention, which may have prevented a future cerebrovascular event. Many physicians perform bedside echocardiograms to assess RV dysfunction to risk stratify patients, but this is not considered critical before catheter-directed interventions if patients are otherwise deemed suitable for this intervention. Clinical practice guidelines currently emphasize a multiparametric evaluation in patients with PE, which means that echocardiography is often paired with cross-sectional imaging and lab biomarkers to finalize an intervention plan [6]. However, a formal echocardiogram is not mandated before catheter-directed intervention, which in our case confirmed the extent and location of the clot, thereby changing the course of management. This highlights the importance of obtaining an echocardiogram before catheter-directed interventions in patients with massive and submassive PE. We also believe that surgical embolectomy may be the only definite and viable option when the risk of stroke and systemic embolization is perceived in this patient population.

## Conclusions

Catheter-directed interventions have transformed the management of massive and submassive PE. However, we presented a case where surgical embolectomy was preferred over catheter-directed interventions with an excellent outcome. In our opinion, surgical embolectomy can potentially prove to be a critical life-saving intervention in this subgroup of patients with acute PE and clot-in-transit/PFO. This not only prevented systemic embolization from clot-breaking but also avoided further paradoxical embolism by surgically closing the PFO, as other clots in the venous system can easily migrate from the right side of the heart through the PFO into the systemic circulation.
